# Axiological-Identitary Collective Action Model (AICAM): A new integrative perspective in the analysis of protest

**DOI:** 10.1371/journal.pone.0218350

**Published:** 2019-06-12

**Authors:** José Manuel Sabucedo, Marcos Dono, Dmitry Grigoryev, Cristina Gómez-Román, Mónica Alzate

**Affiliations:** 1 Departament of Social Psychology, Basic Psychology and Methodology, University of Santiago de Compostela, Santiago de Compostela, Spain; 2 National Research University Higher School of Economics, Moscow, Russian Federation; Middlesex University, UNITED KINGDOM

## Abstract

Current predictive models of collective action have devoted little attention to personal values, such as morals or ideology. The present research addresses this issue by incorporating a new axiological path in a novel predictive model of collective action, named AICAM. The axiological path is formed by two constructs: ideology and moral obligation. The model has been tested for real normative participation (Study 1) and intentional non-normative participation (Study 2). The sample for Study 1 included 531 randomly selected demonstrators and non-demonstrators at the time of a protest that took place in Madrid, May 2017. Study 2 comprised 607 randomly selected participants who filled out an online questionnaire. Structural equation modelling analysis was performed in order to examine the fit and predictive power of the model. Results show that the model is a good fit in both studies. It has also been observed that the new model entails a significant addition of overall effect size when compared with alternative models, including SIMCA. The present research contributes to the literature of collective action by unearthing a new, independent path towards collective action that is nonetheless compatible with previous motives. Implications for future research are discussed, mainly stressing the need to include moral and ideological motives in the study of collective action engagement.

## Introduction

Washington D.C., 28 August 1963. Hundreds of thousands of people take part in the *March on Washington for Jobs and Freedom* as part of a claim for civil rights.

Tunisia, 17 December 2010. A period of protests demanding democratic reform begins against the authoritarian regime of Ben Ali.

Dresden (Germany), 22 December 2014. Thousands of people demand more restrictive immigration policies and to brake the entry of refugees.

Spain, March 8 2018. Hundreds of thousands of women take to the streets of many Spanish cities demanding full equality of rights and living conditions.

These are just a few examples of the collective actions that citizens undertake to attempt to influence political authorities and public opinion. Some aim to gain new social rights, others to resist them. Regardless of its purpose, there are three distinct aspects of protest action: a) the pursuit of a collective and non-individual benefit; b) the attached cost, whether large or small, depending on the society and the moment the action takes place; and c) the fact that they have become normalised political actions in current democratic societies [1].

There has long been an interest in knowing which antecedents encourage participation in this kind of political behaviour. Gamson’s approach [2] is a benchmark for much of the research on this subject; he points out three fundamental frames which promote, justify and legitimise collective action: identity, injustice and efficacy. A later meta-analysis performed by van Zomeren et al. [3] shows the predictive relevance of these frames while highlighting the central role of identity. This gave rise to the development of the Social Identity Model of Collective Action (SIMCA), in which identity affects injustice and efficacy, and all three of identity, efficacy and injustice directly promote collective action.

The relevance of these dimensions toward understanding citizens’ involvement in collective action has gathered wide empirical support. However, the question that needs to be raised is if there are other dimensions, in addition to these three, that may be equally relevant but which, for various reasons, have not received as much attention. Reasons might be that some approaches to social behaviour had not yet been elaborated enough, or they were not central to research at the time Gamson or even van Zomeren and his colleagues performed their research.

We believe that the space for the improvement of the models based on the triumvirate of identity, efficacy and injustice resides in the addition of motives that relate to personal beliefs and moral values. In a regular basis, people seem to disregard potential costs to the point of self-sacrifice in order to act morally [4]. Similarly, we argue that they may be willing to put up with the costs of engagement in collective action regardless of its efficacy as long as the collective action concerns morally relevant values. Therefore, we posit people’s ideology and moral values, and the will act upon them, as key factors behind collective action engagement that can complement and amplify our current understanding of this phenomenon.

Ideology is generally conceptualised as a set of collective beliefs about human behaviour and social organisation. For this reason, it is possible to assume that it will promote collective action (directly or indirectly) through the activation of certain interpretative frames of social reality. Therefore, ideology should be part of the models that analyse this type of political participation.

In the case of morality, in recent years there has been a renewed interest in this issue (for a review of moral psychology see [5]). Morality refers to a vast range of human activities, including politics [6]. People may desire the organisation of society to be arranged according to moral beliefs and values, which address central elements of their self-concept. This reasoning lead to an increased relevance of morals in collective action. In particular, there are two moral constructs that have been used to predict collective action: moral conviction [7] and moral obligation [8].

Ideology and morality not only relate to politics, and therefore to protest as regards political expression, but are also linked to issues such as injustice, identity and political efficacy of collective action as named by Gamson. For this reason, the main objective of the study is to incorporate a new motivational path to a predictive model of collective action. We call such a path axiological, a term derived from axiology, which is the branch of philosophy that studies values. The axiological path is composed by two variables: ideology and moral obligation; and it will be integrated with what is already known about identity, injustice and efficacy. Thus, the contribution of this study is to disentangle the distal and proximal antecedents of the collective actions and add a previously often omitted axiological perspective to the framework of a new collective action model.

### Injustice, efficacy and identity

Gamson’s [2] injustice, identity and efficacy are all part of the new model. In the following, we provide a brief review.

First, there is the injustice variable. Derived from relative deprivation theories, it has been shown to be [9,10] a key antecedent in mobilising citizens [2,3,11]. While relative deprivation constitutes the cognitive dimension of injustice, anger accounts for the emotional side, also known as affective injustice. The theory of cognitive appraisal [12] points out that environmental assessments are associated with emotional reactions. Thus, the perception of injustice might trigger the emotion of anger. In the meta-analysis performed by van Zomeren et al. [3], affective injustice showed greater predictive power regarding political action than cognitive injustice. This is why we incorporate affective rather than cognitive injustice into our model.

Efficacy is another primary antecedent of collective action. As Bandura [13] pointed out at the time, self-efficacy is essential for moving from knowledge to action. Hardly anyone would become involved in activities if they did not believe that it would be of use in achieving the desired objectives. Due to the collective nature of political protest, what is required is group efficacy, not personal efficacy [3,11].

Some operationalisations measure the efficacy of collective action as the immediate achievement of group objectives. However, such short-term success is highly unlikely, except in specific and extraordinary cases where a structure of an extremely favourable political opportunity and a massive mobilisation concur. In most cases, if there is an impact of collective action, it is more long-term. Therefore, efficacy should also be measured in terms of whether action allowed people to move toward their objective. Along these lines, Hornsey et al.’s [14] proposal regarding the measure of efficacy centres on other possible achievements of protest, such as visualising certain problems or raising public awareness. This will be the measure of efficacy used in the model proposed in the current study.

Identity is the third and final classic antecedent of action deriving from the work of Gamson in collective frames [2]. There is large number of studies that demonstrate identity’s key role in understanding political action [11,15,16]. However, identifying with a group may not be enough to motivate political participation, as it has been shown that social movements are mainly linked to a certain kind of social identity, a politicised identity. Politicised identity implies not only identifying oneself with a deprived group but also recognising an external agency as responsible for the group’s grievances and realising that engagement in collective action is the means by which the situation can be reversed [17]. The meta-analysis by van Zomeren et al. [3] also supported politicised identity as a better predictor of collective action than other forms of identification. For this reason, politicised identity, not merely social identity, will be the variable featured in the model.

### The axiological path for collective action

#### Moral conviction or moral obligation?

There are two concepts related to morality which have been shown to be linked to collective action: moral conviction and moral obligation [18,19]. Therefore, it must be decided which one may be more useful for achieving the goals of the present study.

Moral conviction has been defined as a “meta-cognition about a given attitude considered to be grounded in core beliefs about fundamental right and wrong”([20], p. 41). As for moral obligation, it has been defined as the belief that one must act according to one’s own values and principles [19]. According to these definitions, we have concluded that even though both concepts are framed in the realm of morality, they allude to differing aspects. That is, two individuals can feel equally that a moral value is essential for them (i.e., share a moral conviction), while only one of them may come to be more willing to act in accordance with that value (i.e., feel a greater moral obligation). This difference may have implications for the prediction of collective action. Moral obligation represents a motivation to behave in a specific way and is therefore closer to the performance of behaviour than moral conviction. This would make moral obligation a better predictor of collective action.

The role of moral conviction in collective action was analysed by van Zomeren et al. [18]. The results showed that conviction exerts an indirect effect through identity, efficacy and injustice. On the other hand, Sabucedo et al. [19] used both measures of morality (conviction and obligation) in order to predict intention to participate in collective action and real participation. Moral obligation turned out to be a better predictor of both perspectives of collective action than moral conviction. For this reason, moral obligation will be a part of this new proposal for a model of collective action.

#### Ideology

Ideology can be defined as a system of personal, internal and consistent beliefs about the right way of organising society [21]. Therefore, it guides the interpretation of the political context. This implies that ideology provides the criteria to assess what is fair or unfair in a situation and to identify, if need be, those responsible. The response to the offers of mobilisation will depend greatly on this assessment. In this vein, Klandermans [22] states that people are inclined to be involved in those movements that are congruent with their ideology. In view of this, we share the surprise of Jost et al. [23] when they claimed ideology has been underestimated in the explanation of collective action.

The inclusion of ideology in this model is even more relevant if we also consider the introduction of moral obligation. Attaining goals that are considered fair and desirable requires the commitment to act to achieve them. The feeling of moral obligation, of acting according to what one believes, can often help overcome the cost associated with the achievement of certain objectives.

It is necessary to clarify that, in this study, ideology is conceptualised as an ideological self-placement. This approach has been questioned on some occasions by referring to the fact that it is a simplification of what ideology really is [24]. Although this criticism may sound sensible, ideological self-placement has shown to be associated with central aspects of ideology: political values [21,25], party identification [26], and political attitudes and opinions [27]. In addition, the use of the self-placement measure has clear advantages in field studies in which there is little time to interview participants, as it is easily understandable and requires only a brief response.

### Development of the proposed model

**Fig 1:**
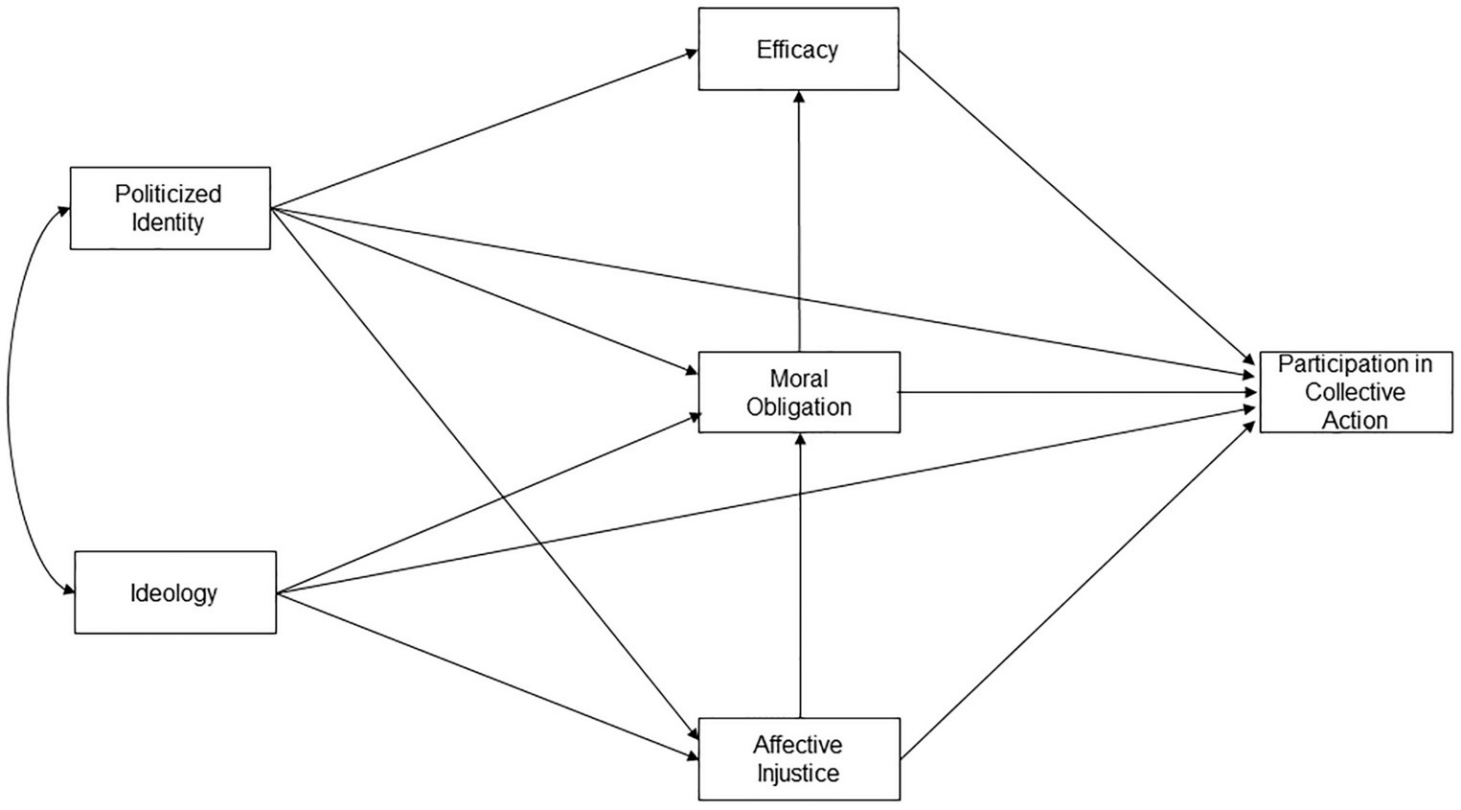
AICAM proposal.

Once the concepts that will be included in the model have been posited and discussed, it is necessary to refer to the hypothesised relations between them (see Fig 1 for a graphical representation of the model). Identity and ideology are posited as the most distal variables of the model, also interacting with each other in a bidirectional way. The feeling of belonging and group identity arises and is strengthened by sharing beliefs, values and objectives [26,28,29]. In the case of politicised identities, a large part of these aspects deal with ideology. For its part, ideology is reinforced, visualises and is sometimes materialised by the action of groups that have formed around it [30]. Hence, the identification with the group is that which allows the existence and transmission of ideology.

As to its link with collective action, it is hypothesised that both should directly predict engagement. The fact both that ideology and identity are the distal variables of the model and that the former is part of the axiological path to collective action is what leads to this proposal being called the Axiological-Identitary Collective Action Model (AICAM).

Identity predicts all three proximal antecedents of the model, namely affective injustice, moral obligation, and efficacy. The relationship of identity with injustice and efficacy was corroborated in a meta-analysis by van Zomeren et al. [3], which gave rise to the SIMCA model. Additionally, identity is also expected to increase the sense of moral obligation to participate among those who share that group’s goal. Moral obligation needs cognitive access to a certain behaviour towards which one should feel obliged. Accordingly, politicised groups provide a set of behaviours (e.g., demonstrating) directed towards defending moral values, which would be unavailable to the individual otherwise. Additionally, the politicised identity makes shared values within the group more accessible and therefore activates the heuristic of availability [31] regarding the issue that motivates collective action. In this way, people are directly challenged to get involved who consider it relevant and also identify with the group, emphasising their feeling of moral obligation. Finally, and related to the explicit demand for participation, we must mention the cost associated with not acting consistently with what is believed [32,33]. The cost is even greater if people identified with the group leading the demand, which therefore provides an example of morally coherent behaviour.

In reference to ideology, it is proposed that it will influence affective injustice and moral obligation but not necessarily efficacy. As previously mentioned, ideology represents a system of attitudes, beliefs and values regarding what social and political life should be like [21]. In this regard, and with respect to injustice, ideology gives us the comparative criteria to evaluate the current socio-political situation (fair vs. unfair, legitimate vs. illegitimate). Ideology thus generates a cognitive evaluation of injustice which in turn generates anger; this way, ideology influences affective injustice. Regarding moral obligation, as the ideology refers to achieving desirable ends, it is reasonable to think that it will influence the moral obligation to act in order to attain them. In a way, both injustice and moral obligation are consequences derived from the very existence of the belief system.

The relationship of ideology with efficacy is not as clear as in the other two cases. Desiring and trying to achieve an objective is not necessarily linked to the perception that it is attainable. Potential participants in a collective action are aware that their demands are presented in an argumentative and political context in which there are other positions, some of which can be totally contrary to theirs. This leads to the question whether ideology can influence efficacy.

As for the predictive role of affective injustice, it is believed to directly influence both the participation in collective action and moral obligation. While its link with participation in collective action is well supported in the literature [3], its effects on moral obligation call for theoretical justification. As mentioned, affective injustice has been mainly identified with the feeling of anger. Functionally, anger has been linked to action readiness in order to facilitate goal-congruent behaviour [34]. Beyond action readiness, anger is also linked with greater tolerance for risk and with disregard of potential costs of action [35]. Therefore, we believe anger will enhance moral obligation mainly through reducing the potential costs associated the particular behaviour towards which moral obligation is felt.

Concerning moral obligation, we hypothesise that it should predict the perception of group efficacy. Here, again, it must be remembered that in this work, we are defending a concept of efficacy that is not limited to the short-term achievement of the goals of protest. While there is no reason to think that a feeling of moral obligation should enhance perceptions of utilitarian efficacy, it may well be related to other conceptions of efficacy, for instance, efficacy in expressing one’s own opinions or in trying to raise public awareness of the issue at stake. Understood in this way, the proposed hypothesis is supported by studies that show that moral motivation is associated with a greater motivation to express values [36] and to attempt to persuade others [37].

It must be noted that this approach in no way undermines the influence of the utilitarian perception of efficacy. We believe that thinking that group goals are attainable promotes engagement in collective action. For us, efficacy and moral obligation represent two independent, compatible paths towards collective action. There may be individuals motivated to participate in a strict utilitarian way, engaging when they perceive a strong chance for success. Conversely, there are other people willing to participate in collective action without thinking that their goals will be met, only for the sake of ‘doing what is right’. Finally, it is possible that in most cases both motivations are present at a variable rate.

In line with the previous argument, these efficacy perceptions and moral obligation are expected to have a direct link with collective action participation, which has been supported by the literature in both instances [3,19].

Two studies were carried out to test the proposed model. Study 1 was performed using a sample of participants at a demonstration that took place in Madrid in May 2017. In the second study, another sample of participants was asked for their intention to participate in non-conventional protest actions.

## Study 1

The objective of this study is to test the AICAM model in the context of a real demonstration. In our opinion, and whenever possible, it is desirable to analyse real collective action and not intention. This helps avoid the well-known behaviour-intention gap (for more details, see [38,39]).

### Method

#### Participants and procedure

The present study was approved by the bioethics committee of University of Santiago de Compostela as it fulfils all demanded ethic requirements, such approval was obtained in written form. Participants were informed about the terms and the general aim of the study by the interviewers present at the demonstration and decided whether or not to give verbal consent. Only those that provided verbal consent went on to complete the questionnaire. The committee can be contacted at: cittinfo@usc.es.

The sample was collected during a demonstration organised by the political party Podemos in Madrid on May 20, 2017. Podemos originated during the 15-M Movement (an anti-austerity movement born in Spain in 2011, for more information see [40]) and is defined as a populist radical-left party [41]. The purpose of the demonstration was to push for a vote of no confidence against the Prime Minister of Spain, at the time Mariano Rajoy.

The sampling procedure was carried out in accordance with the recommendations of [42] with the objective of obtaining a random sample of participants at the rally. Non-participants who were found close to the demonstration were also interviewed. In this way, we made sure participants and non-participants were really who they said they were, given that non-participants, due to proximity, had the possibility to participate but had chosen not to. Data were collected with droidSURVEY software, which was used by the interviewers to register responses. The final sample was 531 participants, of whom 270 were protesters and 261 non-protesters. The mean age was 40.6 years old (*SD* = 15.5), and 273 participants were women (51.4%).

#### Measures

*Moral obligation* was measured using the measure developed by Sabucedo et al. [19]. This measure contained five items with a 7-point Likert scale (ranging from 1 = *totally disagree* to 7 = *totally agree*) (α = .92). An example of an item is ‘*To mobilise for the improvement of labour conditions constitutes a moral obligation to oneself’*.

*Politicised identity* was measured by tapping into the dimensions of cognitive centrality and affective relationship with the reference group [3]. The measure comprised five items with a 7-point Likert scale (α = .96) that measured feelings of sympathy, identification, connection and agreement with the organising groups (e.g., ‘*I share values and beliefs with those who organise the demonstration*’).

*Affective injustice* was measured by two items with a 7-point Likert scale that tapped into how people felt towards the situation (in this case, the government’s recent corruption cases) that was believed by members of the group to be the origin of their grievances (e.g., *angry*, *disgruntled)* (α = .78).

Four items were taken from Hornsey [14] in order to measure *efficacy* perceptions (α = .85). Participants were asked to determine the level of efficacy they perceived, regarding protesting for improved labour conditions and retirement plans, on a Likert scale that ranged from *not effective at all* (1) to *very effective* (5) (e.g., ‘*build an oppositional movement*’).

Finally, *ideology* was measured with an 11-point self-placement left-right scale, where 0 represented *extreme left* and 10 represented *extreme right*.

### Results

#### Preliminary analysis

Those participants who showed loss of data in any of the variables were eliminated. As for the outliers, in this case they were introduced in the analysis without applying previous corrections. The first step in the data analysis was to perform a factor analysis of variance (ANOVA), the factor being the participation variable (participant or non-participant). As expected, the analysis showed significant differences between participants and non-participants in all the variables of the model. Thus, the participants scored higher in all the variables of the model with the exception of ideology, which was to be expected because it was a left-wing demonstration, and the left implies a low score on the ideological self-placement scale (see Table 1).

**Table 1:** Results of intergroup comparisons for the AICAM variables using ANOVA in Study 1 (N = 531).

	*M* (*SD*)		*F*(1)	ω^2^
	Demonstrators (*n* = 270)	Non-demonstrators (*n* = 261)		
Distal antecedents				
Identity	6.18 (1.07)	3.49 (1.84)	430.1	0.45
Ideology	3.12 (1.72)	4.90 (1.82)	133.7	0.20
Proximal antecedents				
Affective Injustice	6.84 (0.56)	6.28 (1.25)	45.6	0.08
Moral Obligation	6.15 (1.07)	3.37 (1.69)	519.6	0.49
Efficacy	3.74 (0.89)	2.71 (0.98)	162.1	0.23

*Note*. All mean differences were significant at *p <* .001.

The bivariate correlations between the variables can be found in Table 2. Regarding the exogenous variables, ideology was negatively associated with moral obligation, affective injustice and efficacy, while identity was positively associated with these variables. At the same time, all of the endogenous variables (moral obligation, affective injustice and efficacy) were positively associated with each other. Only ideology was negatively associated with participation in conventional collective action, while all other variables had positive associations.

**Table 2:** Bivariate correlations between the AICAM variables in Study 1 (N = 531).

	1	2	3	4	5
Distal antecedents					
1. Identity					
2. Ideology	-.55				
Proximal antecedents					
3. Affective Injustice	.37	-.31			
4. Moral Obligation	.75	-.44	.34		
5. Efficacy	.58	-.32	.22	.63	
Outcome					
6. Participation (1 = yes; 0 = no)	.67	-.45	.28	.70	.48

*Note*. All correlations between variables were significant at *p <* .001.

#### Logistic regression

The results of the logistic regression analysis of participation in conventional collective action can be found in Table 3. Overall, the correct classification index amounted to around 87.4%. Both distal antecedents (identity and ideology) provided an odds ratio to participation in conventional collective action, while only moral obligation did so among proximal antecedents.

**Table 3:** Results of logistic regression analysis in Study 1 (N = 531).

	*B*	*SE*	Exp(*B*) [95% *CI*]	*p*	VIF
Distal antecedents					
Identity	0.55	0.11	1.74 [1.41, 2.16]	< .001	1.216
Ideology	-0.31	0.08	0.73 [0.62, 0.86]	< .001	1.051
Proximal antecedents					
Affective Injustice	-0.16	0.17	0.85 [0.60, 1.20]	.354	1.154
Moral Obligation	0.86	0.11	2.37 [1.88, 2.98]	< .001	1.315
Efficacy	0.09	0.16	1.09 [0.79, 1.51]	.598	1.198

*Note*. Log-likelihood = -173.13, χ^2^ = 389.71, *p* < .001, Nagelkerke’s Pseudo-*R*^*2*^ = .69, Cox & Snell Pseudo-*R*^*2*^ = .52

#### Structural equation modelling

The proposed model was tested using structural equation modelling (SEM), which allows for a comparison between the proposed model and alternative models. Since the data contained some non-normal distributed variables, and Mardia’s coefficient was greater than 3 [43], the Satorra-Bentler scaling corrections (MLR) [44] were applied and proved to be a robust method to deal with this kind of data [45]. The *lavaan* R package [46]was used to perform the estimation of the models. Estimates of logit regression were obtained since participation in conventional collective action was a dichotomous outcome.

**Fig 2:**
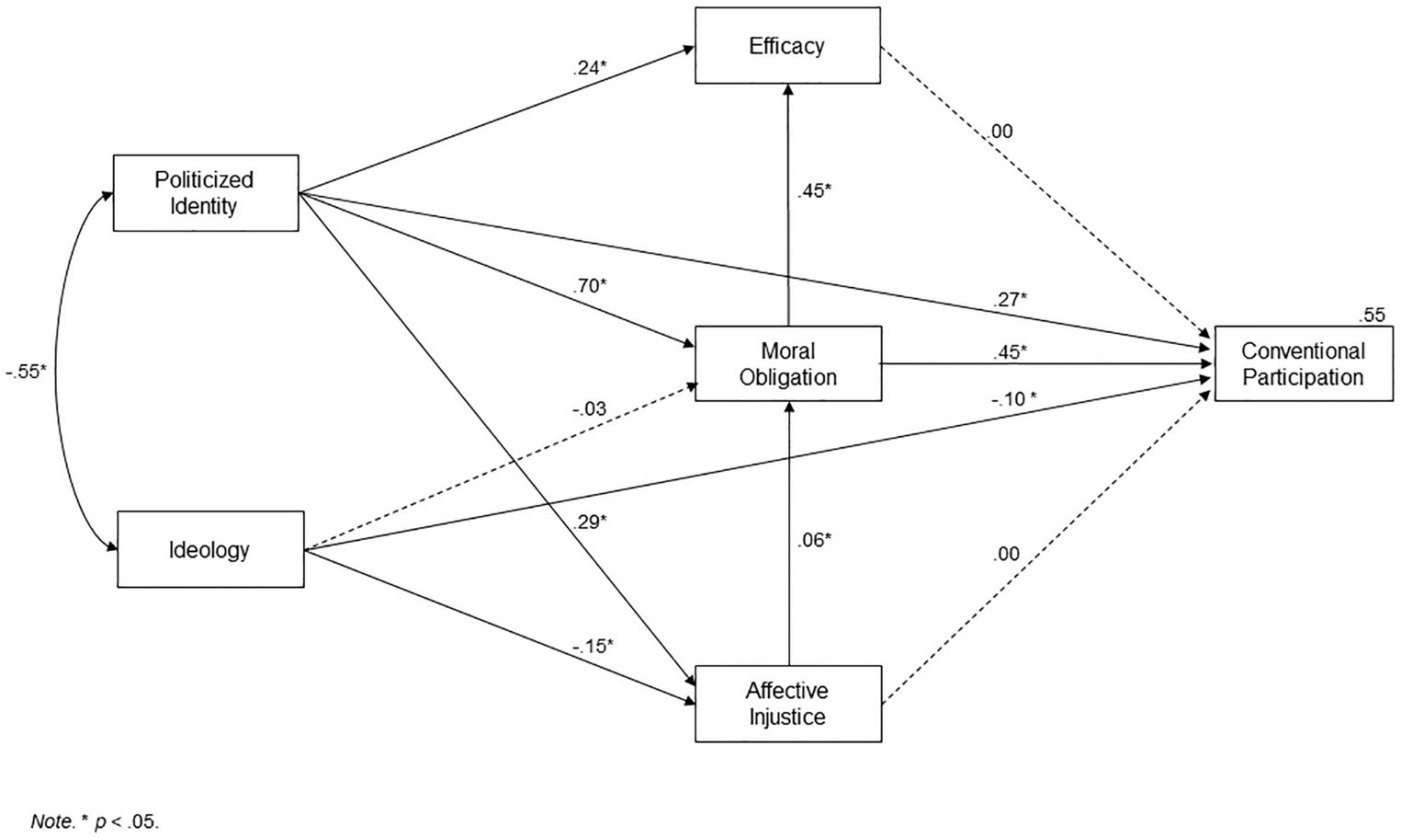
AICAM, Study 1.

The AICAM showed an excellent fit: SB χ^2^ (1, *N* = 531) = 0.434, *p* = .510; CFI = 1.00; RMSEA < .01 with *R*^*2*^ = .55 (see Fig 2 for detailed results).

The goal of the present research was to add an axiological path in a predictive model that also featured the variables of identity, efficacy and injustice. Such variables were successfully integrated into the SIMCA model by van Zomeren et al. (2008). This model should thus be used as a first alternative to analyse whether the introduction of the axiological path into the AICAM represents any improvement. In this case, the SIMCA offered a very similar fit: SB χ^2^ (1, *N* = 531) = 0.066, *p* = .798; CFI = 1.00; RMSEA < .01. As both models had an optimal fit, comparative indices were used in order to decide which one performs better from a statistical point of view.

Accordingly, the AIC and CAIC indices were obtained. These are comparative indices for structural equation models, where the model with the lowest score is the better of the two (Burnham & Anderson, 2003). The AIC index for the AICAM is -3.48, while the CAIC result was -14.03; as for the SIMCA, AIC was -1.92, and CAIC was -7.20. The score for AICAM is lower in both cases; thus, these indices point towards AICAM as the most adequate choice.

Finally, the *R*-squared value of both models was compared. As stated before, the AICAM had *R*^*2*^ = .55 and SIMCA had *R*^*2*^ = .47, which in terms of effect size represents a medium effect (*f*^*2*^ = 0.18) of improvement of the AICAM over the SIMCA.

A second, alternative model was constructed on the basis of a recent serial mediation proposed by [47]. In this work, a model was posited in which moral obligation acted as an antecedent of politicised identity. Accordingly, in this model, ideology should predict moral obligation, which in turn should predict identity. Identity should predict efficacy, affective injustice and participation; then identity, efficacy and affective injustice should predict participation. In this case, the model’s fit was poor (SB χ^2^ (7, 524) = 225.05, *p* = < .001; CFI = .84; RMSEA = .24).

A last alternative model was proposed in which identity was the sole exogenous variable, which predicted affective injustice, efficacy and ideology. Identity was also hypothesised to have a direct link with participation. Ideology predicted moral obligation. Finally, efficacy, injustice and moral obligation all predicted participation. For this model, goodness of fit was not acceptable either (SB χ^2^ (7, 524) = 385.96, *p* < .001; CFI = .73; RMSEA = .32).

### Discussion

The different analyses performed in Study 1 support the AICAM model. The participants in the demonstration obtained the highest scores in all the previous predictor variables—except in ideology, which is also consistent with the model—and the correlation and regression analyses confirm the hypothesised relations between the variables of the model. Along these lines, the analysis of structural equations showed a good fit. More importantly, its fit is better than the proposed alternative models, including the SIMCA, thus representing an improvement with a medium associated effect size (*f*^*2*^ = 0.18).

With regard to assessing a model, especially if it is proposed as an alternative to existing ones, one has to consider other aspects in addition to the statistics. We are referring to whether it offers new insight into the issue being analysed. In our opinion, that criterion is fulfilled with this model. In addition to the traditional paths of injustice, identity and efficacy, the important role of the so-called axiological path (ideology and moral obligation) in collective action is highlighted throughout this study.

As regards more specific aspects of the model, it must be pointed out that not all variables showed a direct and meaningful relationship with participation; this is the case of affective injustice and efficacy. Regarding the former, it is observed that injustice affected participation through its influence on moral obligation. In relation to efficacy, the analyses of hierarchical logistic regression confirm that adding moral obligation to the model caused efficacy to cease to be a significant predictor. The result dovetails with previous work in which both predictors were used [8]. This seems to indicate that in actions where moral obligation is not a determining factor, efficacy can play a more central role, either substituting or complementing it.

Identity once again confirms its place in the dynamics of political protest. It directly predicts participation and has a significant impact on all the other variables that are part of the model.

As for the variables of the axiological path, it is to be noted that ideology predicted all the hypothesised variables except for moral obligation, on which it has an indirect influence through the variables of identity and injustice. Moral obligation was shown to be the best predictor of participation, as both SEM and regression analyses have shown a larger associated effect size.

For Study 1, a sample of participants in a peaceful demonstration was used. As has been said on several occasions, context matters [48,49]. This means that the incidence of the model variables on the behaviour analysed can differ according to different circumstances. Among them, the type of collective action can be one of the most important. For this reason, in Study 2, less conventional collective actions are analysed to test how AICAM works in those conditions.

Such actions pose an increased risk of violent clashes, which is why the possibility of selecting the sample from the participants in these kinds of protests was ruled out. As an alternative, we resorted to asking a sample of people about their intention to participate in this form of political action, a widely used procedure when studying such political behaviour [50].

## Study 2

As mentioned, Study 2 aimed to test the performance of the AICAM model in terms of predicting intention to participate in non-conventional collective action. The data analysis procedures will replicate that of Study 1.

### Method

#### Participants and procedure

The present study was also approved by the bioethics committee of University of Santiago de Compostela as it fulfils all demanded ethic requirements, such approval was obtained in written form.

Participants answered an online questionnaire; distribution and sampling were made through Qualtrics. The sample consisted of 607 participants (304 women), and participants’ mean age was of 45.6. Since the questionnaire was based on intentions and not real participation, a context for the scales was needed. Participants were asked about the situation of the Spanish Health Care System and their willingness to participate in non-conventional collective action in order to defend it.

#### Measures

Participants were asked how much they identified with the ‘white tide’, the movement in defence of Public Health Care in Spain, instead of how much they identified with the organisers of a demonstration (as in Study 1). All other measures corresponded with those of Study 1, with the exception of the intention of non-conventional participation. This measure contained four items with a 7-point Likert (e.g., ‘I would cut off traffic in my city during protest actions’) (α = .88) and was inspired by the work of Sabucedo and Arce [51].

### Results

First, ANOVA as well as correlational tests and linear multiple regression analysis were performed on these data. Results of the ANOVA and correlational tests replicated those of Study 1. However, the results of the linear regression analysis showed different significant predictors of collective action than the logistic regression analysis from Study 1. This time, the effects of identity and efficacy on the intention to participate in collective action were non-significant, whilst affective injustice turned out to be a significant predictor (see Table 4 for a full disclosure of the results).

**Table 4:** Results of linear regression analysis in Study 2 (N = 607).

	B [95% *CI*]	*SE*	β	*p*	*r*_p_^2^ (%)	VIF
Distal antecedents						
Identity	-0.02 [-0.14, 0.10]	0.06	-.02	.723	< 1	2.321
Ideology	-0.13 [-0.20, -0.07]	0.03	-.15	< .001	3	1.229
Proximal antecedents						
Affective Injustice	0.09 [0.01, 0.18]	0.04	.09	.024	1	1.534
Moral Obligation	0.55 [0.42, 0.67]	0.07	.46	< .001	10	2.809
Efficacy	0.02 [-0.14, 0.18]	0.08	.01	.815	< 1	1.536

*Note*. *F*(5) = 63.9, *p* < .001, *R*^*2*^ = .35.

#### Structural equation modelling

**Fig 3:**
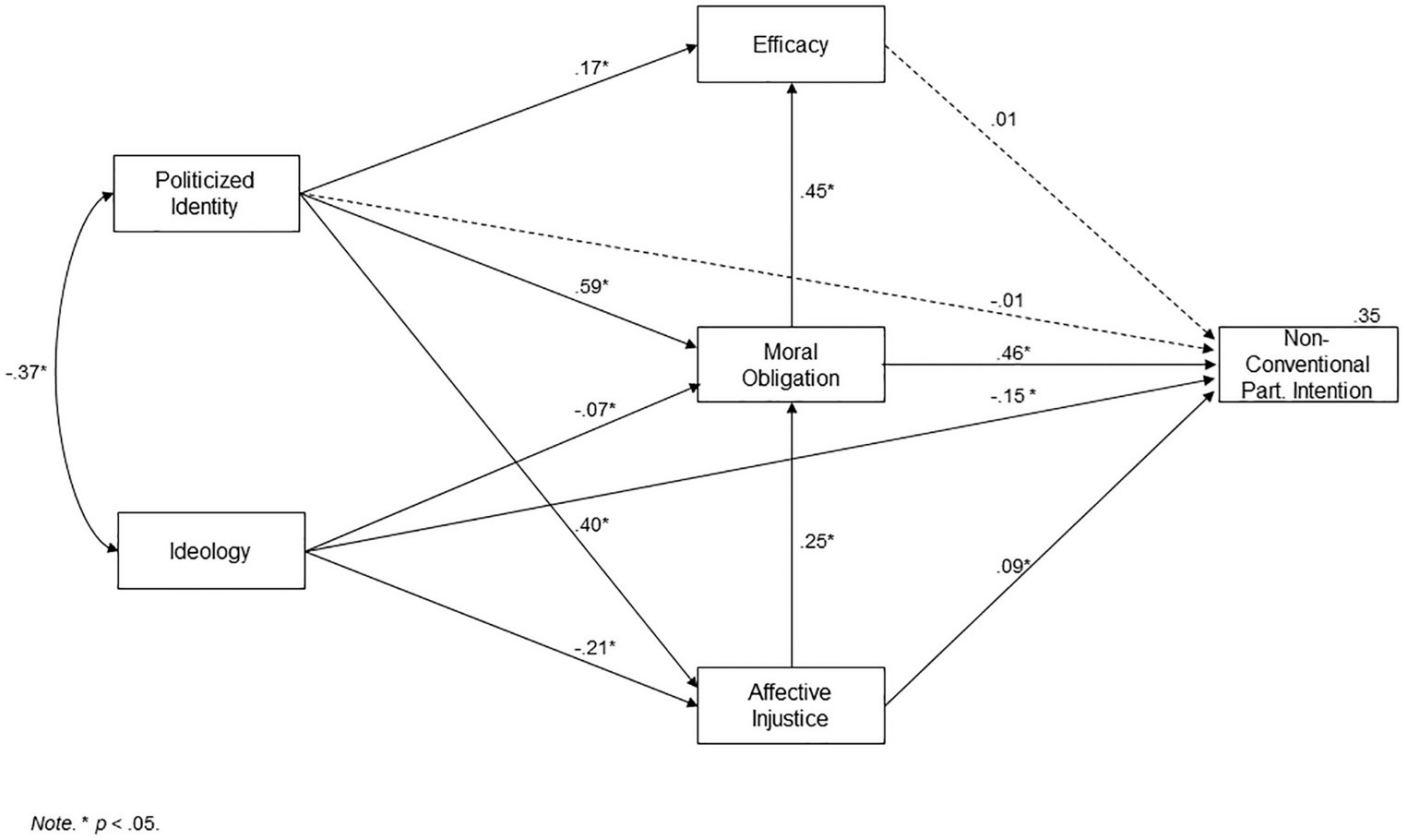
AICAM, Study 2.

In this case, the model showed a similar goodness of fit index to that in Study 1 (SB χ^2^ (2, 605) = 3.72, *p* = .15; CFI = .99; RMSEA = .03), and the level of explained variance was .35 (see Fig 3 for detailed results). The variables that directly predicted intention to participate in non-conventional collective action were ideology, moral obligation and affective injustice.

As for the SIMCA, the fit indices worsened when compared with those of Study 1 SB χ^2^ (1, 606) = 16.56, *p* = < .001; CFI = .96; RMSEA = .16), and the level of explained variance for non-conventional participation was .24. Thus, this time, the increase in explained variance caused by the AICAM variables on the SIMCA was 11%. Moreover, the difference between AICAM and SIMCA, when one looks at the AIC and CAIC, is even bigger than in Study 1 (AICAM AIC = -0.27; SIMCA AIC = 14.56; AICAM CAIC = -11.08; SIMCA CAIC = 9.16).

Alternative Model 2 did not show an acceptable fit (SB χ2 (7, 600) = 178.96, *p* < .001; CFI = .83; RMSEA = .20), and neither did Alternative Model 3 (SB χ^2^ (7, 600) = 435.85, p < .001; CFI = .59; RMSEA = .31).

### Discussion

The good fit shown by the AICAM, with a different sample and type of collective action than that used in Study 1, attest the validity of the model. This assertion is reinforced by the fact that in Study 2, the fit of the AICAM and the variance percentage it explains are also superior to alternative models.

Regarding the variables that had a direct influence on the intention to participate in non-conventional collective actions, we note that in this case, they were ideology, moral obligation (the axiological path proposed in this work) and affective injustice. Of the three, it is moral obligation that again proves it has a greater influence on the intention to participate. In the particular case of intention to participate in non-conventional action, the role of the new axiological path seems even more relevant.

On this occasion, identity does not show a direct relation with the dependent variable; however, this does not diminish its importance in the model, since it affects the other variables, especially moral obligation. As far as efficacy is concerned, there is also no direct impact on the intention to participate. This may also have happened in Study 1, in which the predictive capacity of moral obligation on the dependent variable seems to nullify its incidence.

## General discussion

In this work, two studies were proposed in order to analyse the predictive capacity of a new model (AICAM) of collective action. The novelty of this proposal was to incorporate an axiological path (ideology and moral obligation) into the classic predictors of injustice, identity and efficacy and to separate the distal and proximal antecedents of collective actions according to their place in the nomological network. The obtained results clearly support the explanatory power of the AICAM. The model has been effective in predicting two types of participation (conventional and non-conventional) and using two different operationalisations (real participation and intention). In both cases, the model has shown good rates of fit and explained variance, surpassing the indicators obtained by a proven model such as SIMCA [3].

The limitations of this study are mainly related to the sample. We used only Spanish participants, and the chosen demonstration was organised by a leftist organisation. We did not include actions of other political orientations. In order to test the generalisation of the results found in this study, it would be interesting to check the validity of the AICAM in other countries and with regard to actions of different ideologies.

Regarding morality, this work also supports previous studies [19] showing that moral obligation is a key predictor of collective action. For this reason, it would be advisable for future work to explore not only its psychosocial history but also possible connections to different prosocial behaviours.

The role of ideology is also important to the model due to its direct influence on conventional collective action and on the intention to participate in non-conventional collective action. Even more important may be the role of ideology in predicting some of the model’s proximal antecedents hence making it more robust. However, its incidence may be still greater than that shown in this work. If, instead of using ideological self-placement, we had chosen another measure of ideology closer to the motives that justify collective action, the results could have been even more conclusive. A variable such as system justification [23] could be a good alternative for the one used in this work.

It was unsurprising that a variable such as efficacy was not a significant predictor of collective actions. Efficacy has failed to significantly predict collective action engagement in a number of previous studies [8,19,48]. We believe the reason to be the context in which the protest takes place. For example, the organiser, the mobilisation frame used, the mobilisation goal, the kind of participants (occasional or regular), or the emotional climate, are all factors that can influence which of the motives are activated [49].

Regarding the influence of context, one of AICAM’s main strengths is that it combines utilitarian and moral explanations. Thus, analysing a collective action using AICAM can tell us about the particular importance of each of these two types of motives in a particular. This, combined with the examination of contextual variables can help us determine which scenarios favour collective action framed in moralistic terms, and which ones should be framed as a more practical effort. This information is not only useful for researchers, but it should also be of great interest to activists and other political actors.

The proposed model brings to the fore values and morals as predictors of collective action. In this vein, it joins other approaches [8,19,52,53] stressing that moral values and the obligation to act coherently may help overcome the cost associated with collective action. However, the true novelty and main contribution to the literature is the addition of moral obligation as an entirely new process within the explanatory models of collective action through the so-called axiological path. Moral obligation represents a conscious self-regulatory effort that prompts individuals to behave congruently with their values. As such, it is conceptually different from all other concepts used to explain engagement in collective action. While previous works showed that it was a strong predictor of collective action [19], the present research truly establishes moral obligation as a fully independent process compatible with other motives of collective action present in the literature to this day. This, in conjunction with the fact that moral obligation represents a particularly strong motivator for collective action, calls for its consistent inclusion in the research of collective action motivation. Therefore, we consider that adding this new, independent and powerful process to a coherent explanatory model of collective action is the true differential contribution of this work.

In summary, AICAM introduces a path which appeals to such central elements in people’s lives as principles and values and the obligation to act according to them. Previous models of collective action did not include such motives. However, this did not mean they were not influencing collective behaviour; politics is, among other things, a struggle to impose what Rokeach [54] calls ‘terminal values’, that is, desirable states of existence. AICAM addresses the lack of this elements in previous explanations of collective action by integrating ideology and moral obligation with previous motives. We believe the results of the present study encourage the consistent inclusion of those concepts in forthcoming research that aims to explain the complex phenomenon of collective action engagement.

We are living in a time of change, even of crisis in the Gramscian sense of the word. The Sardinian philosopher and activist said that in times like these, anything can happen. To analyse people’s motives for engaging in movements fighting for change, and for reasons explained above, the AICAM can be a useful tool that presents a new path people may follow into action.

## Supporting information

S1 File Data sheet for Study 1. (SAV)

S2 File Data sheet for Study 2. (SAV)

S3 File Questionnaire example. (DOCX)
