# Supplementary material for: Axiological-Identitary Collective Action Model (AICAM): A new integrative perspective in the analysis of protest
Source: PLoS One. 2019 Jun 12;14(6):e0218350. doi: 10.1371/journal.pone.0218350 (PMC6561589; doi:10.1371/journal.pone.0218350)
Supplement: S3 File — (DOCX) [file pone.0218350.s003.docx]

**Supporting Information.**

**Scales from the research “Axiological-Identitary Collective Action Model (AICAM): A New Integrative Perspective in the Analysis of Protest”.**

**Age**

Please, indicate your age*/Por favor, indique su edad*

**Sex**

Are you… 1) Man; 2) Woman/ *Es usted… 1) Hombre; 2) Mujer*

**Politicized Identity**

“White tide” is the name given to those organizations that jointly mobilize in favor of the public healthcare system. Please, indicate your degree of agreement regarding the following sentences referring to the defense of the public healthcare system./*Se denomina "mareas blancas" al conjunto de organizaciones que se movilizan en defensa de la sanidad pública. Por favor, señale su grado de acuerdo con respecto a las siguientes afirmaciones que hacen referencia a la defensa de la sanidad pública.*

1. I share values and beliefs with the “white tide”/ *Comparto creencias y valores con las “mareas blancas”*
2. I identify with the “white tide”/ *Me siento identificado/a con las “mareas blancas”*
3. I feel affinity for the members of the “white tide”/ *Siento simpatía hacia los miembros de las “mareas blancas”*
4. I see myself as an activist in defense of the public healthcare system/ *Me considero activista en defensa de la sanidad pública.*
5. I feel represented by the “white tide”/ *Me siento representado por las “mareas blancas”*

**Efficacy**

To what extent do you consider that participating in protests in defense of the public healthcare system would be efficacious for…/ *Indique hasta qué punto considera que participar en protestas en defensa de la sanidad pública sería eficaz para…*

1. Influencing politicians in congress/*Influenciar a los politicos en el Congreso*
2. Influencing the public opinion/*Influenciar a la opinion pública*
3. Bulinding an oppositional movement against healthcare cut-offs/*Construir un movimiento de oposición*
4. Expressing certain personal values/*Expresar ciertos valores personales*

**Affective Injustice**

Please indicate to what extent do you feel like that when you think in the management of the healthcare system by the Government:/*Por favor, indique en qué medida se siente de así cuando piensa en la gestión del sistema de sanidad pública llevada a cabo por el gobierno:*

1. Angry/*Enfadado*
2. Disgruntled/*Irritado*

**Ideology**

When talking about politics, people frequently use the expression of left and right winged. Where will you place yourself in a scale where 0 is extreme left and 10 is extreme right?/ *Cuando se habla de política se utilizan normalmente las expresiones de izquierda o derecha. ¿Donde se situaría usted en esta escala donde 0 significa extrema izquierda y 10 extrema derecha?*

**Moral Obligation**

This scale was presented as published in Sabucedo, Dono, Alzate & Seoane (2018); doi: <https://doi.org/10.3389/fpsyg.2018.00418>
